# Inhibition of Oct 3/4 mitigates the cardiac progenitor-derived myocardial repair in infarcted myocardium

**DOI:** 10.1186/s13287-015-0252-5

**Published:** 2015-12-24

**Authors:** Yu Tina Zhao, Jianfeng Du, Youfang Chen, Yaoliang Tang, Gangjian Qin, Guorong Lv, Shougang Zhuang, Ting C. Zhao

**Affiliations:** Department of Surgery, Roger Williams Medical Center, Boston University Medical School, 50 Maude Street, Providence, RI 02908 USA; Department of Medicine, Vascular Biology Center, Medical College of Georgia/Georgia Regents University, 1120 15th Street, Augusta, 30912 GA USA; c, Northwestern University Feinberg School of Medicine, 303 East Chicago Avenue, Tarry 14-725, Chicago, 60611 IL USA; Department of Ultrasound, Second Affiliated Hospital of Fujian Medical University, 40 Zhongshan N Road, Licheng, Quanzhou, Fujian China; Department of Medicine, Rhode Island Hospital, Brown University, 593 Eddy St, Providence, 02903 RI USA

**Keywords:** Oct3/4, Regeneration, Myocardial infarction, Stem cells

## Abstract

**Background:**

Recent evidence has demonstrated that cardiac progenitor cells play an essential role in the induction of angiomyogenesis in infarcted myocardium. We and others have shown that engraftment of c-kit^+^ cardiac stem cells (CSCs) into infarcted hearts led to myocardium regeneration and neovascularization, which was associated with an improvement of ventricular function. The purpose of this study is aimed at investigating the functional role of transcription factor (TF) Oct3/4 in facilitating CSCs to promote myocardium regeneration and preserve cardiac performance in the post-MI heart.

**Methods:**

c-kit^+^ CSCs were isolated from adult hearts and re-introduced into the infarcted myocardium in which the mouse MI model was created by permanent ligation of the left anterior descending artery (LAD). The Oct3/4 of CSCs was inhibited by transfection of Oct3/4 siRNA, and transfection of CSCs with control siRNA serves as control groups. Myocardial functions were evaluated by echocardiographic measurement. Histological analysis was employed to assess newly formed cardiogenesis, neovascularization, and cell proliferations. Terminal deoxynucleotidyltransferase (TdT) nick-end labeling (TUNEL) was carried out to assess apoptotic cardiomyocytes. Real time polymerase chain reaction and Western blot were carried out to evaluate the level of Oct 3/4 in CSCs.

**Results:**

Two weeks after engraftment, CSCs increased ventricular functional recovery as shown by a serial echocardiographic measurement, which is concomitant with the suppression of cardiac hypertrophy and attenuation of myocardial interstitial fibrosis. Suppression of Oct 3/4 of CSCs abrogated functional improvements and mitigated the hypertrophic response and cardiac remodeling. Transplantation of c-kit^+^ CSCs into MI hearts promoted cardiac regeneration and neovascularization, which were abolished with the knockdown of Oct3/4. Additionally, suppression of Oct3/4 abrogated myocyte proliferation in the CSC-engrafted myocardium.

**Conclusion:**

Our results indicate that CSCs-derived cardiac regeneration improves the restoration of cardiac function and is mediated through Oct 3/4.

**Electronic supplementary material:**

The online version of this article (doi:10.1186/s13287-015-0252-5) contains supplementary material, which is available to authorized users.

## Background

Cardiovascular disease (CVD) is the leading cause of deaths worldwide, with myocardial infarction (MI) being associated with significant morbidity and mortality. Despite significant improvements in medical prevention and treatment, the incidence and prevalence of MI-induced heart failure have been increasing steadily [1]. MI often leads to heart failure because of a loss of viable heart muscle and impaired left ventricular function. The lack of regenerative potential of cardiac tissue and insufficient therapeutic options lead to poor prognosis for patients with CVD. The myocardium has long been considered as a terminally differentiated organ because adult cardiomyocytes exhibit little proliferative activity relative to the fetal stage, and until recently it was seen as lacking in regenerative capacity [2, 3]. The adult heart has now been recognized to harbor distinct populations of cardiac progenitors [4], which have the potential to differentiate into cardiomyocytes, endothelia, and vascular smooth muscle cells. Furthermore, several studies have shown that these cardiac progenitor cells are capable of differentiating into cardiac tissue and improving cardiac function after myocardial injury. Exponential advances in stem cell and regenerative biology are beginning to foster a transition towards therapeutic goals for cardiac regenerative medicine [5–7]. Various studies have identified myocardial precursor cells in the postnatal hearts using several different cell surface markers [4, 8, 9]. Many reports have demonstrated that the myocardium harbors a stem cell-like myocyte population with the properties of self-renewal, replication, and clonogenicity [10]. Furthermore, cardiac progenitor cells have been found to have the capacity of differentiating into cardiac tissue and improving cardiac function after a myocardial injury. These new findings led to a novel understanding of both normal and pathologic cardiac developments and homeostasis, and foster a transition towards therapeutic goals for cardiac regenerative medicine. However, poor cell viability, proliferation, and inefficient differentiation following transplantation have limited the reparative capacity of these cells in vivo. Thus, molecular intervention strategies to enhance cardiac stem cell (CSC) proliferation and survival hold dramatic consequences for enhancing myogenesis and will empower a therapeutically relevant implementation of myocardial regeneration. Studies have reported that genetically modified stem cells could repair the infarcted myocardium, prevent remodeling, and nearly normalize cardiac performance [11, 12].

POU family homeodomain transcription factor Oct3/4, also known as POUF51, is expressed in the early stage of embryonic development and plays a pivotal role in regulating lineage progression in embryonic stem cells (ESCs) [13]. Oct3/4 (also termed Oct3, Oct 3/4, or POU5F1) is critical for mammalian embryonic development and for ESC pluripotency [14, 15]. Recent evidence demonstrates that Oct3/4 serves as a transcriptional organizer to modulate pluripotency and differentiation [13]. Together with Sox2 and Nanog, Oct3/4 works as a gatekeeper of pluripotency in ESCs by maintaining its endogenous expression level. In the revolutionary study conducted by the Yamanaka group, Oct3/4 was found to be one of four transcriptional factors that can reprogram differentiated cells into induced pluripotent stem cells (iPSCs) [16, 17]. It also can direct the ESC toward a mesodermal cardiogenic fate. Oct3/4 and Sox17 have been reported to cooperatively drive human pluripotent stem cells into cardiac progenitors [18]. Li et al. [19] demonstrated that formation of Oct3/4–Wnt ternary complexes contributes to the activation of cardiac lineage-related genes through regulating the cardiac lineage factor Mesp1. This evidence indicates that Oct3/4 of stem cells can direct cell pluripotency as well as determining the cell lineage commitment, which is likely dependent on the cell type and experimental models. However, the genetic and epigenetic mechanisms underlying the dual function of Oct3/4, in maintaining pluripotency, or inducing lineage commitment in cardiac progenitor cell-derived regeneration, have yet to be thoroughly investigated. In this present study, we aimed to assess the crucial role of Oct3/4 in the modulation of CSC-derived cardiac repair and neovascularization in cell-engrafted MI hearts. Our results presented here demonstrate that Oct3/4 plays a pivotal role in directing CSCs to promote cardiac repair when introduced into the MI heart.

## Materials and methods

### Animals

All animal procedures were carried out in accordance with the guidelines approved by the Institutional Animal Care and Use Committee of Roger Williams Medical Center. CD-1 mice were purchased from Charles River Laboratories (Wilmington, MA, USA) and housed at Animal Care Facility of Roger Williams Medical Center on a 12-hour light/dark cycle with free access to water and standard mouse food.

### c-kit^+^ CSC isolation, establishment of stable GFP-c-kit^+^ CSCs, and small interfering RNA transfection

c-kit^+^ CSCs were isolated as described previously [20, 21]. Ventricular tissues from 3-month-old male CD-1 mice were minced into small pieces and subjected to enzymatic dissociation with a mixture of 0.2 % trypsin and 0.1 % collagenase IV (Worthington Biochemical Corp., www.worthington-biochem.com) in phosphate-buffered saline (PBS) three times for 5 minutes at 37 °C. After treatment, the remaining tissue fragments were cultured as explants in explant medium (Iscove’s modified Dulbecco’s medium (IMDM) with 10 % fetal calf serum (FBS) containing 100 U/ml penicillin G, 100 μg/ml streptomycin, 2 mmol/l l-glutamine, and 0.1 mmol/l 2-mercaptoethanol) at 37 °C under normal tissue culture conditions. Three weeks after cell inoculation, small phase-bright cells migrating above the fibroblast layer were formed from adherent explants. In order to avoid damaging the integrity of the surface antigen of cells, these phase-bright cells were collected simply by washing with D-Hanks. The cell suspension was filtered through a 40 μm cell strainer (BD Falcon, www.bdbiosciences.com), and cell numbers were counted. The selected CSCs were cultured and maintained in complete media containing DMEM (Dulbecco's Modified Eagle's medium)/F12, 10 % fetal calf serum, 200 mM l-glutamine, 55 nM β-mercaptoethanol, and 1 % MEM (Minimum Essential Medium) nonessential amino acids (Invitrogen Corporation ). To enrich the c-kit^+^ CSCs, c-kit^+^ cells were isolated by magnetic cell sorting with CD117 magnetic beads (Miltenyi Biotec Inc., Auburn, CA, USA) as instructed by the manufacturer’s protocols. To track the fate of transplanted CSCs in the infarcted hearts, a stable c-kit^+^ CSC line expressing green fluorescent protein (GFP) was established. Freshly isolated c-kit^+^ CSCs were transfected with the linearized pEGFP using lipofectamine™ 2000 (Invitrogen Corporation, Carlsbad, CA, USA) and maintained in the presence of G418 (500 μg/l) for 2 weeks. The transfected GFP-c-kit^+^ CSC-positive colonies were formed, picked up under fluorescent microscopy, and expanded for subcultures in the medium already described. To prevent the effects of multiple passages on CSCs, CSCs at passage 4 were used in this study. The method of small interfering RNA (siRNA) transfection was conducted using the lipofectamine™ 2000 transfection kit (Thermo Fisher, Waltham, MA, USA) according to the manufacturer’s instructions. Oct3/4 siRNA (mouse) was used in these studies, which consist of a pool of three different siRNA duplexes (Santa Cruz Biotech, Santa Cruz, CA, USA). The sequences of Oct3/4 siRNA 1 duplexes are as follows: Oct3/4 siRNA sc-36124A, sense 5′-GAAGGAUGUGGUUCGAGUAtt-3′ and antisense 5′-UACUCGAACCACAUCCUUCtt-3′; sc-36124B, sense 5′-GCUCUCCCAUGCAUUCAAAtt-3′ and antisense 5′-UUUGAAUGCAUGGGAGAGCtt-3′; sc-36124C, sense 5′-CCUUCAGGAGAUAUGCAAAtt-3′ and antisense 5′-UUUGCAUAUCUCCUGAAGGtt--3′. The negative control (scrambled) siRNA and Oct3/4 siRNA were mixed with Lipofectamine™ 2000 at a final concentration of 500 nmol/L siRNA in medium, respectively. Forty-eight hours after siRNA transfection, in vivo allogeneic c-kit^+^ CSC transplantation was performed during the left anterior descending coronary artery (LAD) ligation operation in MI mice.

### Myocardial infarction

Adult mice were randomized into four groups: 1) sham group, sham-operated mice; 2) MI-PBS group, mice with MI receiving PBS injection; 3) MI-CSCs-Oct3/4(+) group, mice with MI receiving control siRNA-treated CSC transplantation; 4) and MI-CSCs-Oct3/4(−) group, mice with MI receiving Oct3/4 siRNA-treated c-kit^+^ CSC transplantation. LAD ligation was performed on all groups except on the sham group to induce MI following thoracotomy, as described previously [21]. Briefly, mice were anesthetized with pentobarbital sodium (50 mg/kg) intraperitoneally, intubated, and then ventilated with positive pressure using a ventilator (Harvard Apparatus Inspira, Holliston, MA, USA). Additional doses of pentobarbital were given as needed during the procedure to maintain an anesthetized state. After a left-sided thoracotomy, the hearts were exposed and the LADs were occluded by a permanent ligation using a 7–0 suture (Ethicon, Norderstedt, Germany). Mice in the sham group were anesthetized and underwent thoracotomy without coronary ligation.

### In vivo allogeneic c-kit^+^ CSC transplantation

Upon completion of LAD ligation, a total of 5 × 10^5^ GFP-labeled c-kit^+^ CSCs were suspended in 10 μl PBS and directly injected into five sites in the border zone of the infarcted left ventricle. Mice in the MI-CSCs-Oct3/4(+) group received control siRNA-treated c-Kit^+^ CSCs, while MI-CSCs-Oct3/4(−) mice received Oct3/4 siRNA-treated c-Kit^+^ CSCs. The MI-PBS group received PBS-only injections. Following CSC/PBS injections, the chest was closed in a layered fashion, and air was evacuated to prevent pneumothorax. Myocardial functions were assessed by echocardiographic measurement. At the end of experiment, 5-ethynyl-2′-deoxyuridine (EdU, 50 mg/kg) was intraperitoneally injected into animals 24 hours prior to euthanization for assessment of cell proliferation in vivo.

### Echocardiography

Two weeks after LAD ligation and CSC engraftment, left ventricular (LV) function was evaluated by transthoracic echocardiography using an Acuson Sequoia C512 system (Siemens Healthcare, Malvern, PA, USA) equipped with a 15 L8 linear array transducer. Mice were anesthetized with 1.5 % isoflurane mixed with oxygen via a nose cone, and then placed in supine position on a heating pad. The precordial region was covered with prewarmed ultrasound transmission gel (Aquasonic; Parker Laboratory, Fairfield, NJ, USA) after removal of hair. Two-dimensional short-axis images were obtained and recorded at the level of the mid-papillary muscle in both B-mode and M-mode. The signal depth was set at 25 mm. Three to six consecutive cardiac cycles were measured from M-mode tracings with accompanying software. LV internal dimension in diastole (LVID;d), LVID in systole (LVID;s), LV ejection fraction (LVEF), and LV fractional shortening (LVFS) were calculated as indexes of LV function.

### Immunohistochemistry

Animals were sacrificed after echocardiographic measurements, and tissues were harvested for immunocytochemistry and histological analyses. Hearts were isolated and fixed in 4 % buffered formalin, then dehydrated and embedded in paraffin. Heart sections were cut, mounted, deparaffinized in xylene, and rehydrated through an ethanol series. Paraffin-embedded tissues were analyzed for fibrosis, pathological hypertrophy, and angiogenesis. Interstitial collagen deposition was assessed by picro-sirius red staining. Tissue sections were stained with picro-sirius red solution for 1 hour at room temperature following 8 minutes of hematoxylin staining. After dehydrating the slides, collagen content was determined from five randomly selected regions from each tissue section at 40× magnification using an Olympus BX51 microscope (Olympus, Center Valley, PA, USA). For immunofluorescence staining, antigen retrieval was performed on deparaffinized tissue sections by boiling slides in citrate buffer at 100 °C for 1 hour. Slides then were blocked in 1 % bovine serum for 1 hour at room temperature followed by overnight incubation with primary antibodies at 4 °C. The following primary antibodies were used in this study: newly formed c-kit^+^ CSC-derived myocytes were identified with polyclonal GFP and costained with α-sarcomeric actinin (both Life Technologies, Carlsbad, CA, USA), microvessel densities were examined by anti-α-smooth muscle actin (α-SMA) monoclonal antibody (Sigma, St. Louis, MO, USA), CD31 (anti-PECAM-1; Millipore, Billerica, MA, USA) was used to identify capillaries in myocardium, polyclonal Ki67 for the cycling myocytes (Novocastra, Milton Keynes, UK) was used to detect the cycling cells, and phosphorylated histone 3 (PH3; Abcam, Cambridge, MA, USA) was used to detect mitosis. After washing in PBS, signals were then visualized by incubating at room temperature for 1 hour with secondary antibodies of Alexa Fluor 488 or 555-conjugated goat anti-mouse, anti-rabbit, or anti-rat antibody (Life Technologies) for 1 hour at room temperature. 4,6-Diamidino-2-phenylindole (DAPI; Life Technologies) was used to identify nuclei. Cross-sectional cell area measurements were obtained in cross-sectioned left ventricles and were measured by overnight staining with wheat germ agglutinin (WGA, fluorescein isothiocyanate-conjugated; Life Technologies) on deparaffinized slides at 4 °C. Confocal fluorescent imagining was performed using a Carl Zeiss LSM 700 laser scanning microscope equipped with intuitive ZEN 2009 software (Carl Zeiss Microscopy, Jena, Germany). Five to 10 randomly selected high-power fields were selected for quantifications using ImageJ software (version 1.40 g; NIH, http://rsb.info.nih.gov/ij/). A minimum of 200 myocytes from five different animals was quantified for each experimental group. Global heart architecture was determined by staining with a Trichrome Stain (Masson) Kit (Sigma-Aldrich, St. Louis, MO, USA) following the manufacturer’s instructions. The infarction size was determined and expressed as the ratio of scar length to LV circumferences of the endocardium and epicardium. 

### Terminal deoxynucleotidyltransferase nick-end labeling

Terminal deoxynucleotidyltransferase nick-end labeling (TUNEL) was carried out using a TACS 2-TdT-DAB In Situ Apoptosis Detection Kit (Trevigen Inc., Gaithersburg, MD, USA) following the manufacturer’s instructions, using an 1-hour labeling reaction in the presence of cobalt. To verify that apoptosis occurred in the myocytes, immunohistochemical staining of α-sarcomeric actin was carried out with an α-sarcomeric actin antibody (α-actin, monoclonal, 1:100 dilution; Sigma) at 4 °C overnight. For each section, the number of TUNEL-positive myocyte nuclei was counted in five randomly selected regions. The index of apoptosis was then determined.

### Real-time polymerase chain reaction

Total RNA was extracted from CSCs from different groups with Trizol reagent (Life Technologies, Grand Island, NY, USA). cDNA was synthesized from 5 μg total RNA. The reverse-transcribed cDNA (5 μl) was amplified to a final volume of 50 μl by PCR under standard conditions. Real-time PCR experiments were performed on a master cycler realplex4 (Eppendorf North America, Hauppauge, NY, USA) system using qPCR Kit master mix (Kapabiosystems, Boston, MA, USA) Primer sequences of Oct3/4 used in these studies are as follows: forward, 5′-TCTTTCCACC AGGCCCCCGG CTC-3′; and reverse, 5′-TGCGGGCGGACATGGGGAGATCC-3′. GAPDH was used as the internal control.

### Electrophoresis and western blot analysis

Hearts tissues were homogenized in ice-cold RIPA buffer (Sigma-Aldrich) containing protease inhibitor cocktails (Calbiochem, Billerica, MA, USA). Protein lysates were then obtained after centrifugation at 12,000 × *g* for 15 minutes at 4 °C. Protein concentrations were estimated using a Micro BCA Assay Kit (Thermo Scientific, Rockford, IL, USA). Proteins (50 μg/lane) were separated on reducing SDS polyacrylamide gels and transferred to PVDF membranes. Then 5 % nonfat dry milk was used to block the membranes at room temperature for 1 hour followed by overnight incubation with primary antibodies at 4 °C. The membranes were then incubated in the appropriate horseradish peroxidase-conjugated secondary antibody solution. The blots were incubated with their respective polyclonal antibodies Oct3/4 (Santa Cruz Biotech) and β-actin (1:1000) for 2 hours and visualized by incubation with anti-rabbit horseradish peroxidase-conjugated secondary antibody (1:5000) for 1 hour and developed with ECL Chemiluminescence detection reagent (Amersham Pharmacia Biotech (Amersham, Buckinghamshire, UK).

### Statistical analysis

Data presented in this study are expressed as mean ± standard error of the mean (SEM). Statistical analysis was performed with one-way analysis of variance followed by a Bonferroni correction for multiple comparisons. *p* <0.05 is considered statistically significant.

## Results

### Oct3/4 inhibition prevented c-kit^+^ CSC transplantation from restoring LV function post MI

**Fig. 1:**
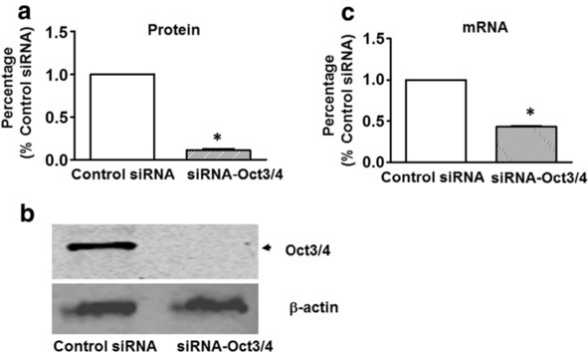
Effect of Oct3/4 siRNA transfection on Oct3/4 expression in c-kit^+^ CSCs. **a** Densitometric analysis showing Oct3/4 protein in cultured CSCs transfected with control siRNA and Oct3/4 siRNA, respectively. **b** Western blot showing the representative gel of Oct 3/4 protein. **c** Real-time PCR showing Oct3/4 mRNA in cultured c-kit^+^ CSCs transfected with control siRNA and Oct3/4 siRNA, respectively (*n* = 3 per group). Values represent mean ± SEM. *p <0.05 vs. control siRNA. *siRNA* small interfering RNA

**Fig. 2:**
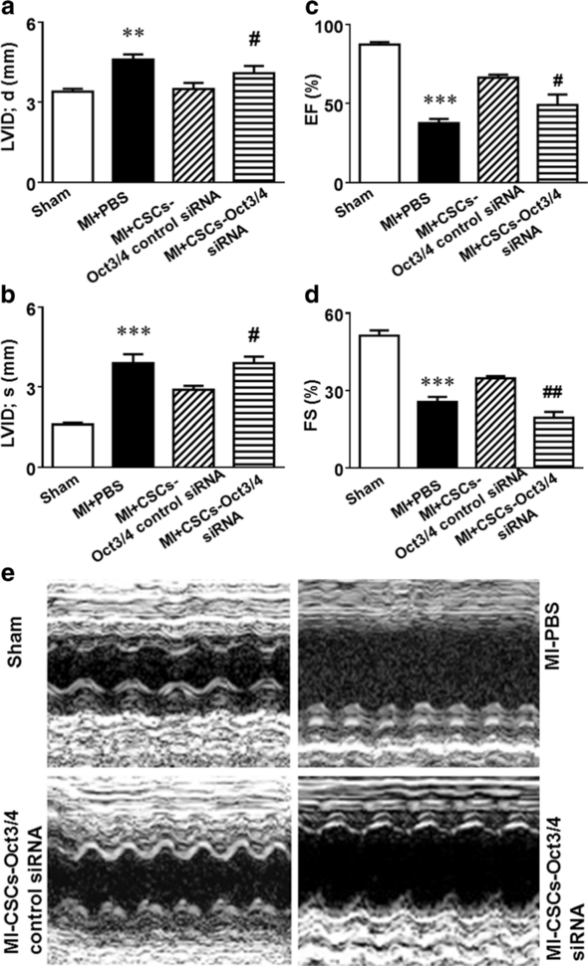
Oct3/4 is required for c-kit^+^ CSC transplantation to improve impaired LV function in post-MI mice. Echocardiographic measurements of LV functional parameters: left ventricular internal dimension in diastole (*LVID;d*) (**a**), left ventricular internal dimension in systole (*LVID;s*) (**b**), ejection fraction (*EF*) (**c**), and fractional shortening (*FS*) (**d**). Representative M-mode images (**e**). Data shown as mean ± SEM (*n* = 5 per group). ***p <0.001 vs. sham, **p <0.01 vs. sham, #*p* <0.05 vs. MI + CSCs-Oct3/4 (+) control siRNA, ##*p* <0.01 vs. MI + CSCs-Oct3/4(+) control siRNA. *CSC* cardiac stem cell, *MI* myocardial infarction, *PBS* phosphate-buffered saline, *siRNA* small interfering RNA

The concentration of Oct3/4 protein and mRNA of CSCs were determined for cell engraftments into MI heart. Knockdown of Oct3/4 significantly reduced Oct3/4 protein and mRNA in CSCs transfected with Oct3/4 siRNA compared with the control siRNA-treated group (Fig. 1). To investigate whether the preservation of LV function by c-kit^+^ CSC transplantation involves Oct3/4, echocardiographic measurements were performed 2 weeks after engraftment. As shown in Fig. 2, an increase in left ventricular internal dimension (LVID) and a significant decrease in contractility were observed in MI-PBS groups as compared with sham-operated mice, indicating a MI-induced LV dysfunction and myocardial remodeling. Both LVID;d and LVID;s were depressed in mice receiving control siRNA-treated CSCs as compared with mice receiving PBS injection post MI (Fig. 2a, b, e), suggesting that CSC engraftment prevented left ventricular dilation in the MI heart, but the LV diameters were not reduced in mice receiving Oct3/4 siRNA-treated CSC injections. Successful engraftment of CSCs prevented systolic dysfunction in MI mice as evidenced by the decreases in ejection fraction and fraction shortening 2 weeks after surgery as compared with the PBS-treated MI group, but in mice that received Oct3/4 siRNA-treated CSCs there was no significant difference observed in LVEF and LVFS as compared with MI-PBS mice (Fig. 2c, d), indicating that Oct3/4-deficient CSCs failed to preserve LV contractility post MI. These results suggest that CSC transplantation restores LV function in the post-MI heart through an Oct3/4-dependent mechanism.

### Inhibition of Oct3/4 suppressed CSC proliferation and cardiac commitments in post-MI myocardium

**Fig. 3:**
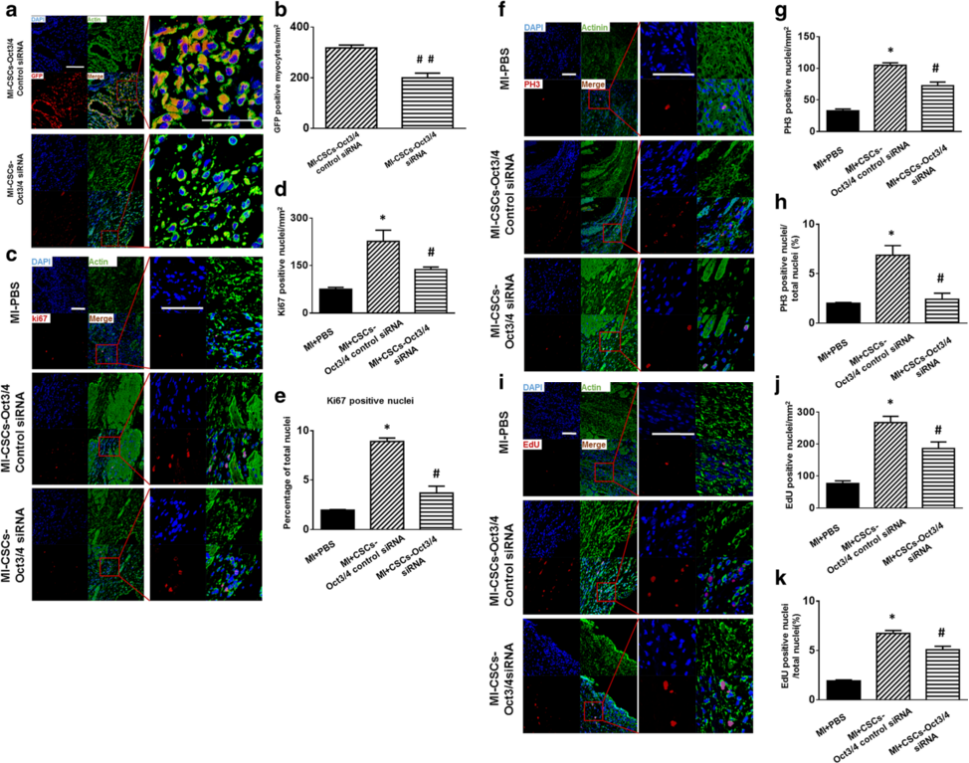
Knockdown of Oct3/4 of CSCs inhibited CSC-derived myocyte proliferation and cardiac commitments in post-MI myocardium. **a** Representative staining images of c-kit^+^ CSC-derived cardiomyocytes. c-kit^+^ CSCs were stained with GFP (*red*); cardiomyocytes were identified with α-actin (*green*). **b** Quantitative analysis of GFP/α-actin positive myocytes. **c**, **f**, ** i** Representative staining images of Ki67/α-actin (**c**), PH3/α-actin (**f**), and EdU/α-Actin (**i**). Cardiomyocytes were identified with α-actin (*green*). Ki67 (*red*), PH3 (*red*), and EdU (*red*) were used to determine myocyte proliferation. **d**, **e**, **g**, **h**, **j**, **k** Quantitative analyses of Ki67, PH3, and EdU staining. The number of positive-stained myocytes was counted in five or six randomly chosen fields per heart. Three to five hearts were analyzed per group. Data shown as mean ± SEM. *p <0.05 vs. MI-PBS, **p <0.01 vs. MI-PBS, #*p* <0.05 vs. MI + CSCs-Oct3/4(+) control siRNA, ##*p* <0.01 vs. MI + CSCs-Oct3/4(+) control siRNA. Scale bar: 100 μm. *CSC* cardiac stem cell, *DAPI* 4,6-diamidino-2-phenylindole, *EdU* 5-ethynyl-2′-deoxyuridine, *GFP* green fluorescent protein, *MI* myocardial infarction, *PBS* phosphate-buffered saline, *PH3* phosphorylated histone 3, *siRNA* small interfering RNA

GFP served as a marker to identify the CSC progeny in cell-CSC engrafted myocardium, and Ki67 was utilized to determine dividing amplifying cells. Mitosis was visualized by PH3, and EdU staining was also carried out to evaluate cardiomyocyte proliferation. The signals for Ki67, PH3, and EdU in the sham group demonstrated rare staining, which is shown in Additional file 1: Figure S1. CSC-derived cardiomyocytes were identified by GFP and α-actinin double-positive staining was located in the infarct sites of CSC-engrafted MI hearts as shown in Fig. 3a, b, but newly formed myocytes were reduced in MI hearts that received Oct3/4 siRNA-treated CSCs. Myocytes in the sham and control MI groups are shown in Additional file 2: Figure S2. Both Ki67-positive and PH3-postive myocytes were augmented in the MI myocardium receiving CSC injections as compared with MI alone, but inhibition of Oct3/4 resulted in reduced CSC-derived Ki67-positive and PH3-positive myocytes as shown in Fig. 3c, d, e, f, g, h. EdU-positive myocytes increased in the MI hearts transplanted with CSCs as compared with MI alone. However, MI hearts engrafted with Oct3/4 siRNA-treated CSCs (Fig. 3i, j, k) attenuated the EdU-positive myocytes, indicating that Oct3/4 plays a pivotal role in CSC-driven cardiomyocyte proliferation. Additionally, there were no differences in cell MEF2C, Ki67, and PH3-positive cells (data not shown), indicating that the short-term knockdown of Oct3/4 could not induce a significant change in cell proliferation nor differentiation. Likewise, there was no significant change in cell phenotype.

### Inhibition of Oct3/4 abrogated c-kit^+^ CSC-derived myocardium regeneration and angiogenesis

**Fig. 4:**
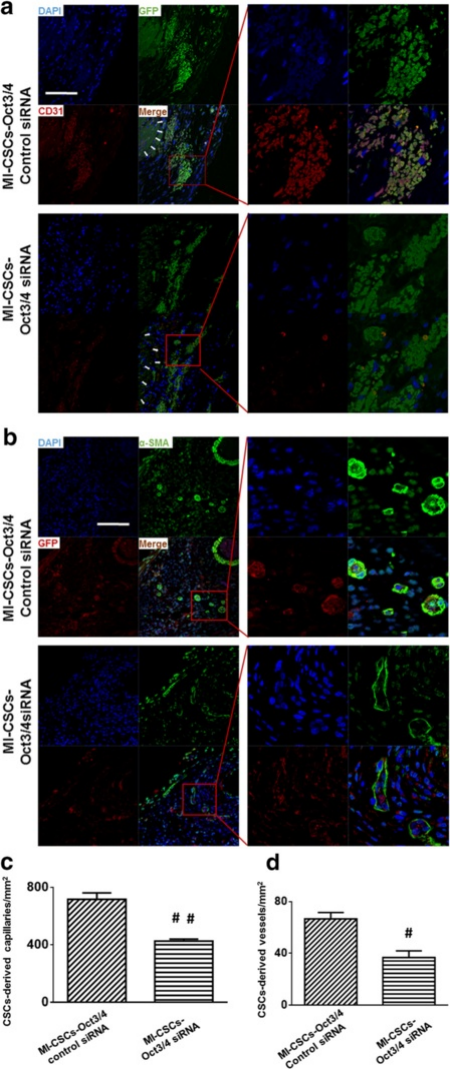
Inhibition of Oct3/4 mitigated CSC-derived myocardium regeneration and angiogenesis in post-MI heart. Representative images of CD31 (**a**) and α-SMA (**b**) staining in post-MI hearts, respectively. **c**, **d** Quantitative analysis of c-kit^+^ CSC-derived angiogenetic response. Results were indicated by the number of CD31-positive or α-SMA-positive cells per mm^2^. Values shown as mean ± SEM (*n* = 4 per group); #*p* <0.05 vs. MI + CSCs-Oct3/4(+) control siRNA, ##*p* <0.01 vs. MI + CSCs-Oct3/4(+) control siRNA. Scale bar: 100 μm. *CSC* cardiac stem cell, *DAPI* 4,6-diamidino-2-phenylindole, *GFP* green fluorescent protein, *MI* myocardial infarction, *siRNA* small interfering RNA

To assess the angiogenic responses in post-MI hearts, we measured the vascular density by immunofluorescent staining of vascular α-SMA and the capillary density using CD31 staining which identifies endothelial cells. Newly formed microvessels were determined with GFP/α-SMA staining, while GFP/CD31 staining was utilized to identify CSC-derived myocardial capillaries. As shown in Fig. 4a, c, inhibition of Oct3/4 resulted in a substantial reduction in the capillary density in the border area of CSC-engrafted infarct heart as compared with MI heart receiving Oct3/4(+) CSCs. α-SMA/GFP staining demonstrated a decrease in CSC-derived microvessels in MI hearts engrafted with Oct3/4 siRNA-treated CSCs (Fig. 4b, d). Both CD31-positive and α-SMA-positive stainings were detectable in sham and MI hearts as shown in Additional file 3: Figure S3. In addition, inhibition of Oct3/4 of CSCs showed a slight increase in apoptotic CSCs in cell-engrafted MI hearts (Additional file 4: Figure S4). Taken together, the data indicate that inhibition of Oct3/4 attenuated CSC-promoted cardiac regeneration and angiogenesis.

### Oct3/4 inhibition attenuated the anti-remodeling effect of c-kit^+^ CSC transplantation in post-MI heart

**Fig. 5:**
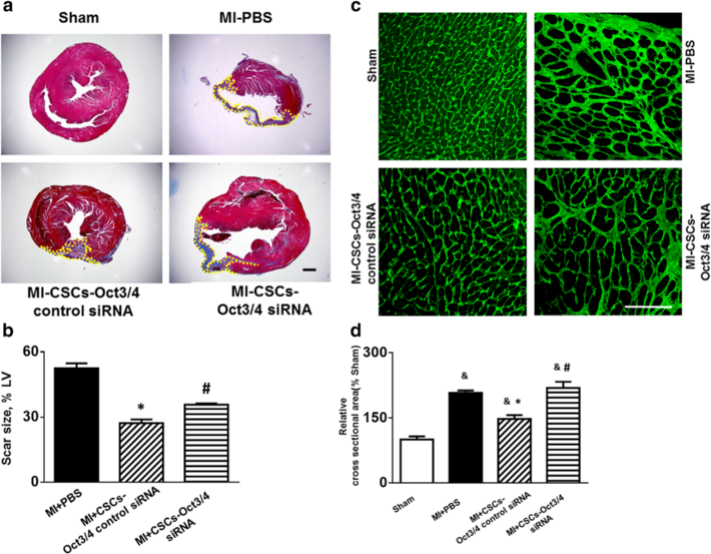
Inhibition of Oct3/4 attenuated the capacity of CSCs to prevent MI-induced cardiac hypertrophy and remodeling. **a** Representative images of Masson’s trichrome-stained myocardial left ventricle in MI hearts receiving c-kit^+^ CSC transplantation and PBS injection. Scale bar: 100 μm. **b** Quantitative analysis of myocardium infarct size. **c** Representative images of WGA staining in post-MI mouse hearts. Scale bar: 100 μm. **d** Quantitative analysis of myocyte cross-sectional area. Data shown as mean ± SEM (*n* = 3–5 per group). &*p* <0.05 vs. sham, *p <0.05 vs. MI-PBS, #*p* <0.05 vs. MI + CSCs-Oct3/4(+) control siRNA. *CSC* cardiac stem cell, *LV* left ventricular, *MI* myocardial infarction, *PBS* phosphate-buffered saline, *siRNA* small interfering RNA

Masson’s trichrome staining was used to access morphometric changes in the post-MI heart. As shown in Fig. 5a, staining images of MI hearts engrafted with Oct3/4(+) CSCs demonstrated a more viable myocardium with smaller scar size, indicating that CSC transplantation can successfully prevent post-MI cardiac remodeling. Suppression of Oct3/4 of CSCs failed to prevent the MI-induced remodeling (Fig. 5b). WGA staining was performed to measure the size of cardiomyocytes close to the infarct border. As shown in Fig. 5c, d, MI led to a significant increase in cross-sectional cardiomyocyte diameters in PBS-treated mice as compared with mice in the sham group. Oct3/4(+) CSC transplantation led to a significant reduction in cross-sectional cardiomyocyte diameters compared with MI-PBS mice, but inhibition of Oct3/4 abrogated the beneficial effect of CSC treatment of MI heart, suggesting that the anti-remodeling process of CSC treatment in post-MI heart involves Oct3/4.

### Inhibition of Oct3/4 increased apoptosis and fibrosis in c-kit^+^ CSC-engrafted MI heart

**Fig. 6:**
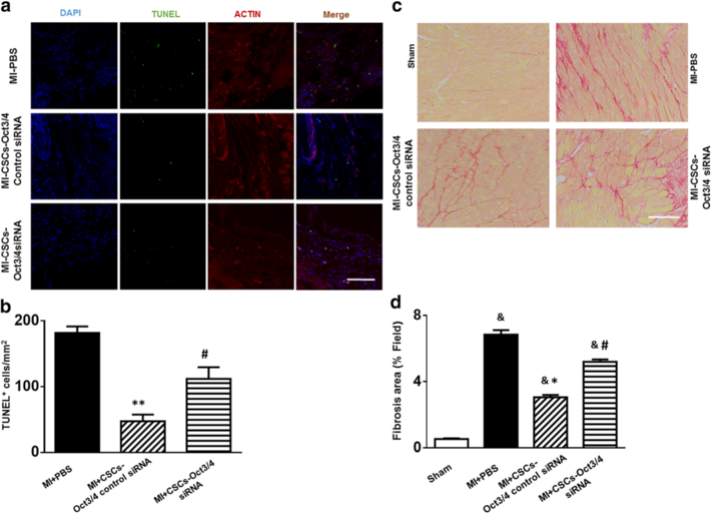
Inhibition of Oct3/4 attenuated the anti-apoptotic and anti-fibrotic effects of engrafted CSCs in post-MI heart. **a** Representative images of TUNEL staining (*green*, TUNEL-positive staining; *red*, α-actin; *blue*, DAPI). Scale bar: 100 μm. **b** Quantitative analysis of TUNEL-positive nuclei. **c** Representative images of picro-sirius red staining. Scale bar: 50 μm. **d** Quantitative analysis of interstitial collagen deposition in post-MI hearts. Values shown as mean ± SEM (*n* = 3–5 per group); &*p* <0.05 vs. Sham, *p <0.05 vs. MI-PBS, **p <0.01 vs. MI-PBS, #*p* <0.05 vs. MI + CSCs-Oct3/4(+) control siRNA. *CSC* cardiac stem cell, *DAPI* 4,6-diamidino-2-phenylindole, *MI* myocardial infarction, *PBS* phosphate-buffered saline, *siRNA* small interfering RNA, *TUNEL* terminal deoxynucleotidyltransferase nick-end labeling

TUNEL assay was carried out to determine apoptotic myocytes in post-MI heart. A significant reduction in TUNEL-positive myocytes was observed in the infarct sites of the Oct3/4(+) CSC-engrafted myocardium, but the anti-apoptotic effect of CSC transplantation was partially attenuated by Oct3/4 suppression as shown in Fig. 6a, b. Interstitial collagen deposition accompanied by MI in the mouse heart was evaluated with picro-sirius red staining. The collagen content was significantly increased after MI in mice receiving PBS injection as compared with the sham-operated group (Fig. 6c). Oct3/4(+) CSC transplantation resulted in a decrease of interstitial collagen in response to MI, which was abolished in hearts receiving Oct3/4 siRNA-treated CSCs, as the collagen content remained significantly elevated in those groups (Fig. 6d), indicating that the prevention of post-MI apoptosis and fibrosis by CSC transplantation is dependent on the participation of Oct3/4.

## Discussion

Recent developments in stem cell research provide new approaches in treating MI-induced heart failure focusing on replacing the damaged myocardium with viable cardiomyocytes. Cardiac progenitor cells are shown to have beneficial effects in preventing negative remodeling processes and restoring cardiac function in post-MI hearts [21, 22]. However, the molecular mechanism(s) by which CSCs exert myogenesis and cardiac regeneration still need to be elucidated. Genetic interventions are widely applied to induce knockdown of specific genes in progenitor cells [23]. In this present study, we observed that knocking down the Oct3/4 gene using a siRNA transfection assay abolished beneficial effects of c-kit^+^ CSC engraftment in post-MI mouse heart.

The level of Oct-3/4 expression in ESCs is crucial in order to maintain their stem cell characteristics, including pluripotency. Oct3/4 acts in a gene dose-dependent manner as a transcriptional organizer in a complex network of tissue specific transcriptional factors in the earlier stages of stem cell and blastocyst differentiation [13]. A recent observation uncovered key roles of Oct-3/4 in ESC specification towards a cardiac lineage and in mesodermal commitment of the embryonic epiblast [24]. cDNA antisense-mediated and siRNA-mediated inhibition of upregulation of Oct-3/4 in ESCs prevents their specification towards the mesoderm and their differentiation into cardiomyocytes. Furthermore, Oct-3/4 siRNA injected into the inner cell mass of blastocysts impairs cardiogenesis in early embryos [25]. In our study, specific inhibition of Oct3/4 in CSCs resulted in the reduction of cardiac functional improvements as compared with control siRNA, which suggests the essential role of Oct3/4 in CSCs to affect c-kit^+^ CSC-derived myocardial regeneration and functional restoration. In addition, hearts in the Oct3/4 siRNA CSC-treated group demonstrated much greater amounts of accelerated cardiac remodeling and functional dysfunction. The mechanism is not clear but may be related to the duration of MI. In agreement with our observations, the previous study demonstrates that valproic acid, an HDAC inhibitor, enhances the ability of miniature pig somatic cell nuclear transfer embryos to develop into blastocysts and maintains their ability to express the Oct3/4 gene [26]. This is supported by reports [18, 24] which concludes that Oct3/4, in association with Sox2 and Sox17, works as a key switch in determining the cardiogenic fate of stem cells. Infarcted hearts receiving Oct3/4 siRNA-treated CSCs did not demonstrate a formation of tumorigenesis 6 months after cell engraftment (data not shown), suggesting that Oct3/4 inhibition in c-kit^+^ CSCs does not increase the risk of developing teratomas. It was reported that the turning on of the Sox17 promoter will initiate a powerful process to generate a subset of endoderm-expressing Sox17 to regulate paracrine signals for cardiogenesis, including a release of BMP2 into the environment [18] to induce cardiac specification [27]. It is not clear whether Oct3/4 also directly mediates cardiac transcriptional factors to direct the fate of cell specification.

Post MI, the left ventricle undergoes a series of architectural and structural changes along with accelerated fibrosis and collagen deposition, consequently leading to contractile dysfunction and ultimately heart failure [28, 29]. We and others have reported previously that engraftment of stem cells significantly preserved LV function and prevented cardiac remodeling in the murine heart following LAD ligation-induced MI [20, 21, 30, 31]. In this study, we demonstrated the anti-remodeling effect of CSC engraftments in which LV hypertrophic changes featuring enlargement of LV chambers, increased LV weight, and increased individual myocyte size were partially prevented by injection of CSCs into MI myocardium. Post-MI hearts injected with Oct3/4 siRNA-treated CSCs did not show the beneficial effects of CSCs as they demonstrated the hypertrophic response and cardiac remodeling. The mechanisms of Oct3/4 of CSCs for the suppression of cardiac remodeling are not clear. Considering that attenuation of cardiac remodeling was not attributable to survival in the myocardium, it is likely that paracrine effects of CSCs following inhibition of Oct3/4 may play a role in the event [32]. It is also interesting to study whether the differences in engrafted cell numbers also impact the magnitude of cardiac remodeling, which could provide interesting information.

Neovascularization was significantly increased in MI hearts receiving c-kit^+^ CSCs, but the enhanced angiogenesis and atherogenesis were specifically inhibited in Oct3/4 siRNA-treated CSCs, suggesting that Oct3/4-mediated inhibition in microvessels in CSC-engrafted hearts might also be responsible for the improvement of myocardial function and the reduction of myocardial remodeling. This is supported by observations that the augmentation of neovascularizations in MI hearts was closely associated with the prevention of cardiac remodeling [33, 34]. In this study, the knockdown of Oct3/4 of c-kit^+^ CSCs exhibited a decrease in newly formed myocytes in CSC-engrafted MI hearts. Post-MI remodeling is attributed partially to accelerated fibrosis to an extent in the right ventricular and LV myocardium caused by excess pathological collagen deposition [35]. In this present study we demonstrated that the genetic knocking down of Oct3/4 in c-kit^+^ CSCs eliminated the anti-fibrotic effect of CSC transplantation in the post-MI heart. This is supported by both our and other previous reports showing that CSCs reduced the post-MI fibrotic process and reduced infract size [21, 30, 31, 36], suggesting that the anti-remodeling effect of CSCs in cell-engrafted MI hearts is also mediated through a Oct3/4-dependent cellular mechanism. The increase in survival of myocytes in stem cell-engrafted MI hearts has been extensively observed in experimental in vivo animal models. Notably, our finding demonstrates that injection of CSCs reduced apoptotic myocytes in MI heart, but inhibition of Oct3/4 abrogated the effect of transplanted CSCs on reducing myocyte apoptosis. Since the apoptotic cardiomyocytes within infarcted and remote areas were detected in this observation, it is likely that both surviving and newly formed cardiomyocytes could be subjected to apoptosis during an ischemic environment. We have demonstrated previously that engrafted CSCs decreased gradually following engraftment as a result of the hypoxic environment of MI. Although the function of Oct3/4 of CSCs is most contributable for modulating cell proliferation and differentiation, we also found that inhibition of Oct3/4 resulted in a slight increase in apoptotic signals in cell-engrafted MI hearts. Nevertheless, it is not clear whether the cardiomyocyte survival rate is also modulated by Oct3/4, which is limited in this study and merits further investigation into the effects of Oct3/4 on stem cells. Furthermore, we did not specifically estimate the time course of cell survival following cell engraftment. Some of the engrafted cells decreased because of cell death in the hypoxic myocardium, while other engrafted cells underwent cell differentiation. It would be interesting to estimate cell death and survival using the time course of cell engraftment in infarcted heart, which merits further investigation.

## Conclusions

Taken together, our results indicate that transplantation of cardiac progenitors promoted functional restoration, attenuated cardiac hypertrophy, and suppressed remodeling in CSC-engrafted MI hearts. However, these protective effects of CSCs on functional improvement and anti-hypertrophic response in the MI heart were diminished by knockdown of Oct3/4 of CSCs. Inhibition of Oct3/4 of CSCs mitigated the CSC-derived cardiac lineage commitment and proliferation following reintroduction into the MI heart. Furthermore, the increased neovascularization in CSC-engrafted MI myocardium was also attenuated by knockdown of Oct3/4 of CSCs. CSC transplantation in the post-MI heart demonstrated an anti-apoptotic effect in MI hearts, which was also inhibited by knockdown of Oct3/4 of CSCs. Our results demonstrate that Oct3/4 plays a crucial role in mediating CSCs to induce cardiac regeneration. Our study not only provides new insights into our understanding of myocardial repair, but also holds promise in developing a novel therapeutic approach for cardiac regeneration.

## Additional files

Additional file 1: Figure S1. Showing immunostaining signals for Ki67, phosphorylated histone-3 and EdU from sham myocardium. Representative immunostaining images from sham hearts. α-Actin (*green*) was used to stain cardiomyocytes. Ki67 (*red*), PH3 (*red*), and EDU (*red*) were used to examine myocyte proliferation. (Staining results from other groups are presented in Fig. 3f–k.) Scale bar: 100 μm. The detailed procedure is described in Materials and methods. (TIF 365 kb) Additional file 2: Figure S2. Showing negative controls for GFP signals from Sham and MI myocardium. Representative negative control immunostaining images of c-kit^+^ CSC-derived cardiomyocytes. GFP (*red*) was used to identify c-kit^+^ CSCs, and cardiomyocytes were stained with α-actin (*green*). Only images from sham and MI-PBS groups are presented. Scale bar: 100 μm. The detailed procedure is described in Materials and methods. (TIF 350 kb) Additional file 3: Figure S3. Showing immunostaining signals for CD31 and α-SMA from sham and MI myocardium. Hearts from sham and MI + PBS groups were stained with CD31 (*red*) and GFP (*green*) to determine the development of c-kit^+^ CSC-derived capillaries. c-kit^+^ CSC-derived vascular smooth muscle cells were stained with α-SMA (*green*) and GFP (*red*). (Positive staining of c-kit^+^ CSCs from other groups are presented in Fig. 4a, b) Scale bar: 100 μm. The detailed procedure is described in Materials and Methods. (TIF 295 kb) Additional file 4: Figure S4. Showing apoptotic signals from CSC-engrafted myocardium. A Representative immunostaining images of apoptosis in post-MI myocardium. Cardiomyocytes that underwent apoptosis were identified by active caspase-3 (*green*). CSC-derived cardiomyocytes were stained with GFP (*red*). DAPI was used to visualize nuclei. Scale bar: 50 μm. B GFP-positive apoptotic cells in MI heart that received CSC engraftment. The unit was normalized to the cell number per mm^2^. The primary antibodies that were used in this study include the following: engrafted CSCs were identified with mouse monoclonal GFP (Clontech, Mountain View, CA, USA) and costained with polyclonal active caspase 3 (1:100; Abcam, Cambridge, MA, USA). The methodology for immunostaining is identical to the described description in Materials and methods. Value represent mean ± SE (*n* = 3 hearts per group). (ZIP 271 kb)

## Abbreviations

α-SMA: anti-α-smooth muscle actin
CSC: Cardiac stem cell
CVD: Cardiovascular disease
DAPI: 4,6-Diamidino-2-phenylindole
EdU: 5-Ethynyl-2′-deoxyuridine
ESC: Embryonic stem cell
FBS: Fetal calf serum
GFP: Green fluorescent protein
IMDM: Iscove’s modified Dulbecco’s medium
iPSC: Induced pluripotent stem cell
LAD: Left anterior descending coronary artery
LV: Left ventricular
LVEF: Left ventricular ejection fraction
LVFS: Left ventricular fractional shortening
LVID;d: Left ventricular internal dimension in diastole
LVID;s: Left ventricular internal dimension in systole
MI: Myocardial infarction
PBS: Phosphate-buffered saline
PH3: Phosphorylated histone 3
SEM: Standard error of the mean
siRNA: Small interfering RNA
TUNEL: Terminal deoxynucleotidyltransferase nick-end labeling
WGA: Wheat germ agglutinin
