# Supplementary figures and images for: Inhibition of Oct 3/4 mitigates the cardiac progenitor-derived myocardial repair in infarcted myocardium
Source: Stem Cell Res Ther. 2015 Dec 24;6:259. doi: 10.1186/s13287-015-0252-5 (PMC4690244; doi:10.1186/s13287-015-0252-5)

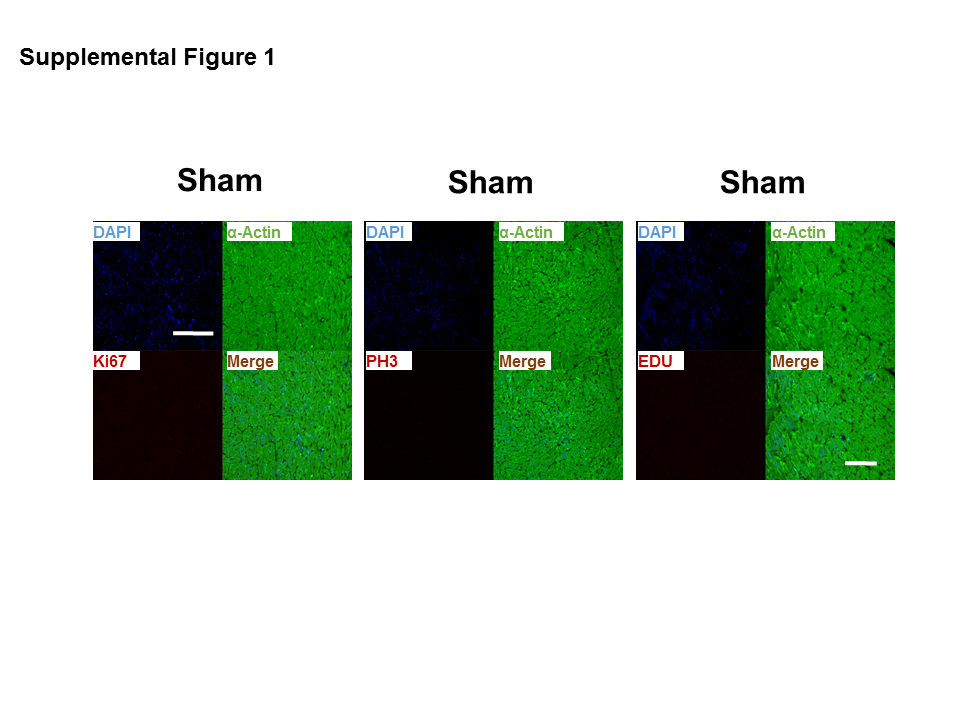

Supplement: Additional file 1: Figure S1. — Showing immunostaining signals for Ki67, phosphorylated histone-3 and EdU from sham myocardium. Representative immunostaining images from sham hearts. α-Actin (green) was used to stain cardiomyocytes. Ki67 (red), PH3 (red), and EDU (red) were used to examine myocyte proliferation. (Staining results from other groups are presented in Fig. 3f–k.) Scale bar: 100 μm. The detailed procedure is described in Materials and methods. (TIF 365 kb) [file 13287_2015_252_MOESM1_ESM.tif]

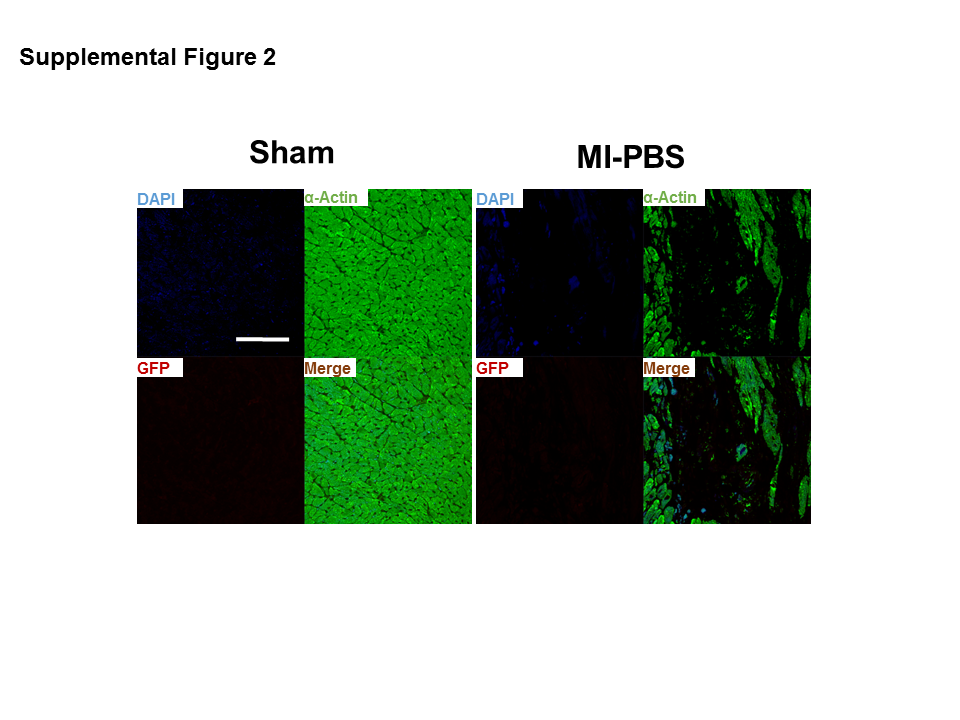

Supplement: Additional file 2: Figure S2. — Showing negative controls for GFP signals from Sham and MI myocardium. Representative negative control immunostaining images of c-kit+ CSC-derived cardiomyocytes. GFP (red) was used to identify c-kit+ CSCs, and cardiomyocytes were stained with α-actin (green). Only images from sham and MI-PBS groups are presented. Scale bar: 100 μm. The detailed procedure is described in Materials and methods. (TIF 350 kb) [file 13287_2015_252_MOESM2_ESM.tif]

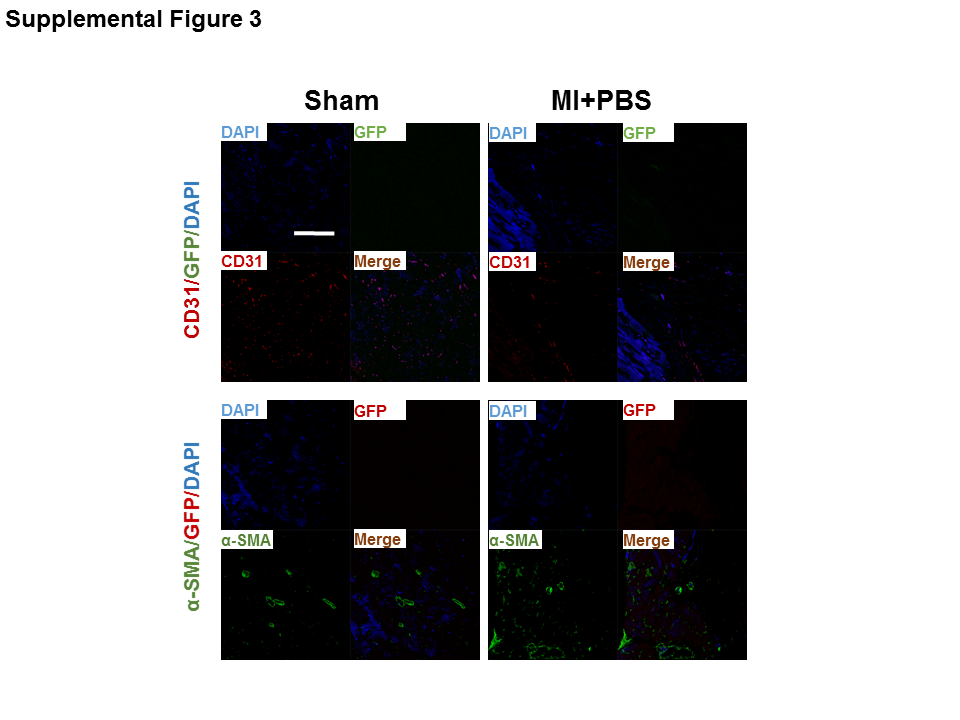

Supplement: Additional file 3: Figure S3. — Showing immunostaining signals for CD31 and α-SMA from sham and MI myocardium. Hearts from sham and MI + PBS groups were stained with CD31 (red) and GFP (green) to determine the development of c-kit+ CSC-derived capillaries. c-kit+ CSC-derived vascular smooth muscle cells were stained with α-SMA (green) and GFP (red). (Positive staining of c-kit+ CSCs from other groups are presented in Fig. 4a, b) Scale bar: 100 μm. The detailed procedure is described in Materials and Methods. (TIF 295 kb) [file 13287_2015_252_MOESM3_ESM.tif]

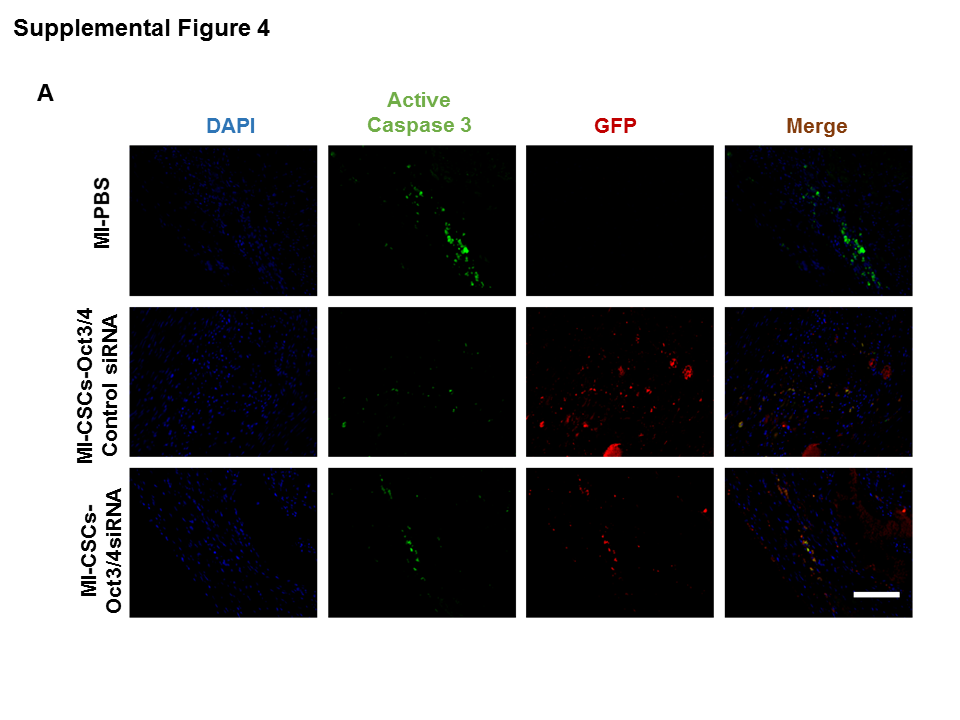

Supplement: Additional file 4: Figure S4. — Showing apoptotic signals from CSC-engrafted myocardium. A Representative immunostaining images of apoptosis in post-MI myocardium. Cardiomyocytes that underwent apoptosis were identified by active caspase-3 (green). CSC-derived cardiomyocytes were stained with GFP (red). DAPI was used to visualize nuclei. Scale bar: 50 μm. B GFP-positive apoptotic cells in MI heart that received CSC engraftment. The unit was normalized to the cell number per mm2. The primary antibodies that were used in this study include the following: engrafted CSCs were identified with mouse monoclonal GFP (Clontech, Mountain View, CA, USA) and costained with polyclonal active caspase 3 (1:100; Abcam, Cambridge, MA, USA). The methodology for immunostaining is identical to the described description in Materials and methods. Value represent mean ± SE (n = 3 hearts per group). (ZIP 271 kb) [file 13287_2015_252_MOESM4_ESM.zip › Supplemental Figure 4A.tif]

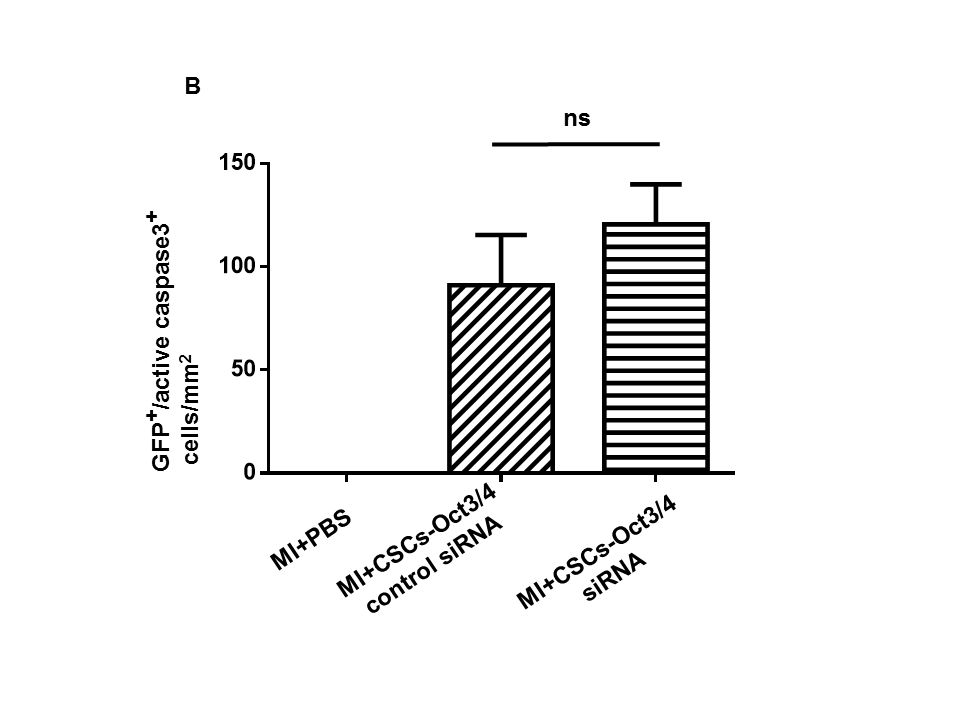

Supplement: Additional file 4: Figure S4. — Showing apoptotic signals from CSC-engrafted myocardium. A Representative immunostaining images of apoptosis in post-MI myocardium. Cardiomyocytes that underwent apoptosis were identified by active caspase-3 (green). CSC-derived cardiomyocytes were stained with GFP (red). DAPI was used to visualize nuclei. Scale bar: 50 μm. B GFP-positive apoptotic cells in MI heart that received CSC engraftment. The unit was normalized to the cell number per mm2. The primary antibodies that were used in this study include the following: engrafted CSCs were identified with mouse monoclonal GFP (Clontech, Mountain View, CA, USA) and costained with polyclonal active caspase 3 (1:100; Abcam, Cambridge, MA, USA). The methodology for immunostaining is identical to the described description in Materials and methods. Value represent mean ± SE (n = 3 hearts per group). (ZIP 271 kb) [file 13287_2015_252_MOESM4_ESM.zip › Supplemental Figure 4B.tif]
